# Fructose Metabolism and Cardiac Metabolic Stress

**DOI:** 10.3389/fphar.2021.695486

**Published:** 2021-06-29

**Authors:** M. Annandale, L. J. Daniels, X. Li, J. P. H. Neale, A. H. L. Chau, H. A. Ambalawanar, S. L. James, P. Koutsifeli, L. M. D. Delbridge, K. M. Mellor

**Affiliations:** ^1^ Department of Physiology, Faculty of Medical and Health Sciences, University of Auckland, Auckland, New Zealand; ^2^ Oxford Centre for Diabetes, Endocrinology and Metabolism, Radcliffe Department of Medicine, University of Oxford, Oxford, United Kingdom; ^3^ Department of Physiology, School of Biomedical Sciences, University of Melbourne, Melbourne, VIC, Australia

**Keywords:** carbohydrate metabolism, diabetic cardiomyopathy (DCM), fructolysis, cardiac lipogenesis, advanced glycation end – products

## Abstract

Cardiovascular disease is one of the leading causes of mortality in diabetes. High fructose consumption has been linked with the development of diabetes and cardiovascular disease. Serum and cardiac tissue fructose levels are elevated in diabetic patients, and cardiac production of fructose via the intracellular polyol pathway is upregulated. The question of whether direct myocardial fructose exposure and upregulated fructose metabolism have potential to induce cardiac fructose toxicity in metabolic stress settings arises. Unlike tightly-regulated glucose metabolism, fructose bypasses the rate-limiting glycolytic enzyme, phosphofructokinase, and proceeds through glycolysis in an unregulated manner. *In vivo* rodent studies have shown that high dietary fructose induces cardiac metabolic stress and functional disturbance. *In vitro,* studies have demonstrated that cardiomyocytes cultured in high fructose exhibit lipid accumulation, inflammation, hypertrophy and low viability. Intracellular fructose mediates post-translational modification of proteins, and this activity provides an important mechanistic pathway for fructose-related cardiomyocyte signaling and functional effect. Additionally, fructose has been shown to provide a fuel source for the stressed myocardium. Elucidating the mechanisms of fructose toxicity in the heart may have important implications for understanding cardiac pathology in metabolic stress settings.

## Introduction

The prevalence of insulin resistance and type 2 diabetes has increased considerably in the last few decades (Ford et al., 2002). It is estimated that currently more than 425 million people globally have diabetes, with numbers projected to increase to ∼700 million by 2045 (Cho et al., 2018). Diabetic patients have an increased risk of developing cardiovascular disease and heart failure (Nichols et al., 2001; Lind et al., 2014; Tancredi et al., 2015). Diabetic cardiomyopathy constitutes a distinct cardiac pathology, independent from other cardiovascular disorders (Huynh et al., 2014; Delbridge et al., 2016; Ritchie and Abel, 2020). Population studies have demonstrated that excess fructose consumption is linked with the development of diabetes and cardiovascular disease (Malik et al., 2010; Johnson et al., 2013; DiNicolantonio et al., 2015; Stanhope, 2016). There is some controversy about whether this association is due to increased energy consumption, or partially mediated by the unique pathological effects of fructose on organ systems (Stanhope, 2016). In pre-clinical rodent studies, isocaloric high dietary fructose induces cardiac metabolic stress and functional disturbance (Mellor et al., 2010b; Mellor et al., 2011b, Mellor et al., 2012). High dietary fructose has also been shown to induce early signs of diastolic dysfunction, alter expression of mitochondrial, apoptotic and oxidative stress-related proteins, and lead to accumulation of cardiac lipid species (Axelsen et al., 2010; Szűcs et al., 2019). Clinically, both circulating and cardiac fructose levels are elevated in diabetic patients (Kawasaki et al., 2002; Daniels et al., 2021), suggesting that direct myocardial exposure to fructose is heightened in a diabetic setting. Pathological outcomes of cardiomyocyte fructose exposure have been demonstrated in tightly controlled *in vitro* experimental settings (Zhang et al., 2016a; Zhao et al., 2017; Fan et al., 2018) and evidence is emerging to support a case for fructose as a pathological agent in the heart. Under normal conditions fatty acids are the predominant cardiac metabolic substrate for ATP supply, and the heart displays remarkable metabolic flexibility in using other substrates such as carbohydrates, amino acids and ketones in response to metabolic stress (Kolwicz et al., 2013). *In vitro* studies suggest that cardiomyocytes have the capacity to metabolize fructose (Mellor et al., 2011a) but the extent to which the heart utilizes fructose as an energy substrate in physiological or pathological settings has not been established. This review aims to survey the clinical and experimental literature assessing fructose involvement in cardiac energy management, cardiomyocyte signaling and metabolism. The potential molecular mechanisms underlying a role for increased fructose exposure in the development of cardiac metabolic stress are examined.

## Sources of Cardiac Fructose

Fructose is a naturally occurring ketohexose sugar (i.e., containing a ketone and six carbons) that is commonly found in fruits or vegetables, where it can either exist alone in its free form or in combination with glucose in the form of sucrose (Rumessen, 1992). Additionally, sweeteners such as high fructose corn syrup have contributed to increased fructose consumption (Pereira et al., 2017). In the heart, cellular fructose content may be determined by the extent of fructose uptake from the systemic circulation and/or by intracellular fructose production via polyol (sorbitol) pathway flux. Tissue fructose levels are typically measured using enzymatic assay or mass-spectrometry based approaches (Kashiwagi et al., 1992; Daniels et al., 2021). Our recent study provided the first evidence that cardiac tissue fructose and sorbitol levels are significantly elevated in type 2 diabetic patients (Daniels et al., 2021). Similarly, cardiac fructose levels have been reported to be elevated in experimental rodent models of both type 1 and type 2 diabetes (Kashiwagi et al., 1992; Lal et al., 1997; Li et al., 2008). These findings suggest that elevated cardiac fructose content and metabolism may be an important characteristic of diabetic cardiomyopathy.

### Extracellular Sources of Cardiac Fructose

Increased circulating fructose has been reported in diabetes, with diabetic patients exhibiting 50–80% increased serum fructose concentration relative to healthy subjects (Kawasaki et al., 2002; Gul et al., 2009). Dietary fructose is absorbed across the intestinal brush border via the facilitative glucose transporter (GLUT)-5 (Burant et al., 1992; Drozdowski and Thomson, 2006). Low level supply of dietary fructose (<0.2 g/kg) is almost completely (∼90%) metabolized into glucose, lactate and glycerate in intestinal cells (Jang et al., 2018). High level dietary fructose (≥1 g/kg) saturates intestinal clearance resulting in spillover into the portal vein via basolateral GLUT2 transporters (Leturque et al., 2005; Douard and Ferraris, 2008; Jang et al., 2018). Portal vein fructose is then taken up by hepatocytes via GLUT2 and GLUT5, where it is predominantly metabolized into glucose and lactate in the liver (Sun and Empie, 2012). However human isotope-labeling studies have demonstrated that a significant proportion of ingested fructose escapes splanchnic extraction (∼15% of a 30 g fructose load) and enters the systemic circulation (Francey et al., 2019). Several fructose transporters such as GLUT5, GLUT11, and GLUT12 have been detected in tissues such as the heart, skeletal muscle, brain, adipose tissue and others (Mellor et al., 2010a). Reported values of fructose levels in the systemic circulation vary widely, likely due to differences in methodologies used to measure fructose, with enzymatic assays often generating higher values than mass spectrometry-based approaches. Clinical guidelines have not been established for defining the “normal” range of human blood fructose levels and literature values for healthy subjects vary widely. Human fasted serum fructose levels are typically reported between 5 and 400 µM (Kruszynska et al., 1993; Tran et al., 2010; Le et al., 2012) and values as high as 1900 µM have been reported in healthy subjects (Hui et al., 2009). Clearance of fructose by the small intestine and the liver appears to maintain baseline fructose concentrations predominantly at the sub-millimolar level, but post-prandial serum fructose levels have been shown to increase into the millimolar range following acute dietary interventions (Kruszynska et al., 1993; Hui et al., 2009; Low et al., 2018). Dietary-derived fructose may therefore play a significant role in determining fructose spillover into the systemic circulation leading to significant cardiac fructose exposure and potential cardiomyocyte fructose uptake, particularly in the context of diabetes.

The mechanisms and kinetics of cardiomyocyte fructose uptake are not fully elucidated. Cardiac expression of fructose transporters, GLUT5, GLUT11 and GLUT12 has been reported (Mellor et al., 2010a, Mellor et al., 2011a; Daniels et al., 2021). GLUT11 and GLUT12 transport glucose and fructose in a competitive manner. Given that circulating millimolar glucose levels exceed reported fructose levels in the micromolar range, GLUT11 and GLUT12 are unlikely to play a significant functional role in cardiomyocyte fructose uptake (Rogers et al., 2002, Rogers et al., 2003; Scheepers et al., 2005). Additionally, the lack of mouse orthologue for GLUT11 has limited investigations using genetic manipulation tools. In contrast, the GLUT5 transporter is highly specific for fructose with a low affinity for glucose transport (Kayano et al., 1990; Burant et al., 1992). Thus, GLUT5-mediated fructose transport is likely to occur even in the context of relatively high circulating glucose concentrations. Whether fluctuations in blood glucose levels influence cardiomyocyte fructose uptake and metabolism, has not been investigated. Emerging evidence suggests that cardiac GLUT5 expression is upregulated in disease states, demonstrated by increased GLUT5 mRNA expression in type 2 diabetic Zucker diabetic fatty rat cardiac tissue (Daniels et al., 2021), and in left-ventricular biopsies from aortic stenosis and hypertrophic cardiomyopathy patients (Mirtschink et al., 2015). In non-cardiac cells, fructose exposure has been shown to increase GLUT5 expression (David et al., 1995; Jiang et al., 2001; Patel et al., 2015) indicating that GLUT5 can be positively regulated by its own substrate. Upregulated fructose uptake in the diabetic heart may contribute to increased intracellular cardiomyocyte fructose, and further investigation is warranted.

### Intracellular Sources of Cardiac Fructose

**FIGURE 1:**
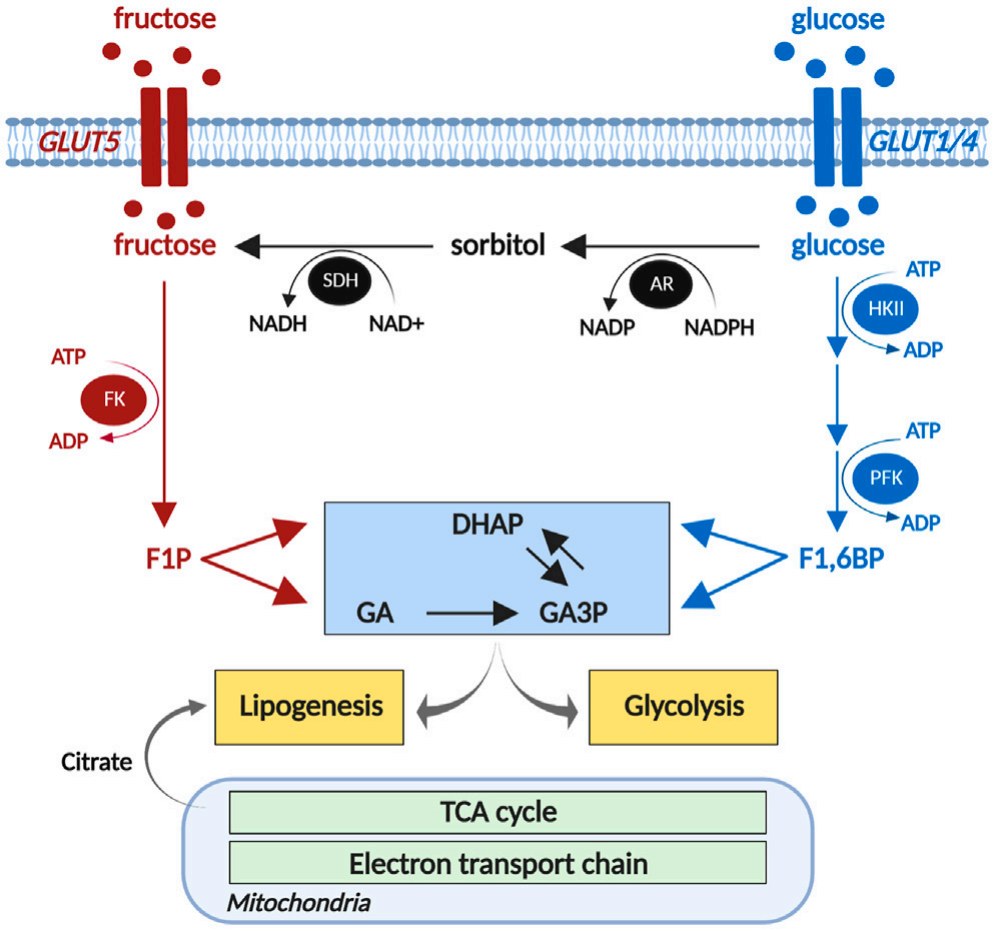
Cardiomyocyte fructose metabolism. Fructose is phosphorylated by fructokinase (FK) to fructose 1-phosphate (F1P), and metabolized to triose phosphates, dihydroxyacetone phosphate (DHAP) and glyceraldehyde (GA). The polyol pathway (black) converts glucose to sorbitol via aldose reductase (AR), and sorbitol to glucose via sorbitol dehydrogenase (SDH). DHAP and GA are converted to glyceraldehyde 3-phosphates (GA3P) and provide substrates for glycolysis to produce acetyl-CoA to enter the mitochondrial TCA cycle. DHAP and TCA cycle-derived citrate contribute to lipid biosynthesis. HKII, hexokinase 2; PFK, phosphofructokinase; TCA, tricarboxylic acid. Created using biorender.com. Adapted from Daniels et al., 2021.

An alternative (non-dietary) source of cardiac fructose is derived from endogenous fructose production via the polyol pathway. In this process, glucose is metabolized into sorbitol via aldose reductase, which is then converted to fructose by sorbitol dehydrogenase (Figure 1; Tang et al., 2012). Cardiac capacity to convert glucose to fructose has been demonstrated in *ex vivo* rat hearts, where 2 h of high glucose (33 mM) perfusion significantly increased cardiac fructose levels (Tang et al., 2010). We have recently shown that cardiac sorbitol levels are increased in human type 2 diabetic hearts (Daniels et al., 2021), consistent with similar findings in type 1 and type 2 diabetic rodent models (Kashiwagi et al., 1992; Lal et al., 1997; Li et al., 2008). Variable findings have been reported regarding the expression of aldose reductase in diabetic hearts. Increased aldose reductase expression is evident in spontaneous type 2 diabetic BBZ rat hearts at 48 weeks of age (Li et al., 2008), contrasting with decreased aldose reductase expression in type 1 diabetic mouse hearts at 3 weeks post-streptozotocin administration (Iwata et al., 2007). These differences may in part be due to the diabetic models used and duration of diabetes. Despite the disparities evident in protein expression, aldose reductase enzyme activity appears to be consistently elevated in type 1 diabetic rodent hearts (Iwata et al., 2007; Sharavana et al., 2017). Sorbitol dehydrogenase protein expression is increased in spontaneous type 2 diabetic BBZ rat hearts at 48 weeks of age (Li et al., 2008), and increased enzyme activity has been reported in type 1 diabetic (streptozotocin-induced) rat hearts (Sharavana et al., 2017). These studies suggest that cardiac fructose production via the polyol pathway may be upregulated in diabetic settings. This finding appears paradoxical in the context of impaired cardiomyocyte glucose uptake and availability due to insulin resistance in type 2 diabetes or insulin deficiency in type 1 diabetes (Desrois et al., 2004; van den Brom et al., 2009). Interestingly, cardiac sorbitol content is correlated with diastolic dysfunction in type 2 diabetic patients (Daniels et al., 2021), suggesting that elevated polyol pathway flux may present an early risk factor for the progression of diabetic cardiomyopathy. High glucose-induced cardiac contractile dysfunction and oxidative stress were ameliorated by inhibition of the polyol pathway enzymes, aldose reductase and sorbitol dehydrogenase (Tang et al., 2010). There is some indication that systemic inhibition of the polyol pathway has favorable cardiac outcomes *in vivo*, with oral administration of an aldose reductase inhibitor (Zopolrestat) leading to improvement in left ventricular ejection fraction in diabetic patients over 1 year of treatment (Johnson et al., 2004). Whether these cardiac effects are secondary to changes in other tissues is yet to be determined.

## Fructose Metabolism in the Heart

The capacity of the heart to metabolize fructose was first observed using isolated adult rat cardiomyocytes, where fructose, in the absence of glucose, sustained cardiomyocyte excitation-contraction coupling acutely (Mellor et al., 2011a). In cultured neonatal rat ventricular myocytes, 1 mM fructose exposure for 24 h significantly increased glycolytic capacity (Daniels et al., 2021). Cellular fructose metabolism is initiated by phosphorylation of free fructose by fructokinase (also known as ketohexokinase) to form fructose 1-phosphate (Figure 1). Fructose 1-phosphate is metabolized to dihydroxyacetone phosphate (DHAP) and glyceraldehyde (GA) which can enter the glycolytic pathway payoff phase (Khitan and Kim, 2013; Mirtschink and Krek, 2016). Unlike tightly regulated glucose metabolism, fructose bypasses the rate-limiting glycolytic enzyme, phosphofructokinase (Khitan and Kim, 2013), and proceeds through glycolysis in an unregulated manner. Experimental upregulation of fructokinase (via genetic splicing) increased fructose metabolic flux in cultured neonatal mouse cardiomyocytes, as evidenced by increased fructose-derived fructose 1-phosphate (Mirtschink et al., 2015). Increased fructokinase expression is evident in Zucker type 2 diabetic rat hearts (Daniels et al., 2021) and in ventricular biopsies of aortic stenosis or hypertrophic cardiomyopathy patients (Mirtschink et al., 2015). Collectively these studies provide evidence that cardiomyocytes have the capacity to upregulate fructose metabolism in both diabetic and heart failure settings.

Fructose-derived glycolytic metabolism generates pyruvate, a well-known substrate of the tricarboxylic acid cycle (TCA) in the mitochondria. Thus it is likely that disturbances in fructose metabolism may have downstream impact on oxidative metabolism. Impaired cardiac mitochondrial function is a common feature of diabetic cardiomyopathy (Diamant et al., 2003; Boudina et al., 2005; Anderson et al., 2009; Pham et al., 2014), but no studies to date have directly investigated whether diabetic mitochondrial dysfunction is linked to cardiac fructose metabolism *in vivo*. *In vitro* evidence suggests that increased fructose exposure induces alterations in cardiomyocyte mitochondrial metabolism. Neonatal rat ventricular myocytes cultured in 25 mM fructose for 48 h exhibit increased mitochondrial complex I and II hydrogen peroxide production (Zhang et al., 2016b), consistent with inefficient mitochondrial electron transport chain function. In H9c2 cardiomyoblasts, 50 mM fructose exposure for 24 h significantly decreased mitochondrial respiration, and ATP production, and increased proton leak (Park et al., 2018). In contrast, mitochondrial function was unchanged in cultured neonatal rat ventricular myocytes exposed to physiological levels (1 mM) of fructose for 24 h (Daniels et al., 2021), suggesting that fructose influence on cardiomyocyte mitochondrial function may only be evident at supra-physiological fructose concentrations. In a setting of enhanced fructose metabolism induced by fructokinase-C overexpression, relatively low-level fructose exposure (25 μM fructose) suppressed mitochondrial oxidative phosphorylation in neonatal mouse cardiomyocytes (Mirtschink et al., 2015). This finding suggests that in a setting of upregulated fructose metabolism, even low levels of fructose may be detrimental to cardiomyocyte mitochondrial function.

## Pathological Outcomes of Cardiomyocyte Fructose Exposure

### Fructose-Induced Cardiomyocyte Lipid Accumulation

Lipid accumulation is a key feature of the diabetic heart (Regan et al., 1977) and is considered to be pathologic. Experimentally, a high fructose diet increases cardiac triacylglycerol (TAG) synthesis and induces cardiac TAG accumulation (Faeh et al., 2005; Stanhope et al., 2009; Fan et al., 2018). Fructose has the potential to promote cardiomyocyte lipid production either as a substrate or via upregulation of enzymes involved in *de novo* lipogenesis (Mirtschink et al., 2015; Fan et al., 2018; Lalowski et al., 2018). Incubating primary mouse cardiomyocytes in [^3^H]-labeled fructose overnight led to direct incorporation of fructose-derived [^3^H] in lipids (Mirtschink et al., 2015), supporting the contention that fructose is a lipid substrate in the heart. Two pathways of fructose metabolism may lead to *de novo* lipogenesis. Citrate produced from fructose-derived DHAP metabolized through glycolysis and subsequent TCA cycle, may be metabolized to fatty acid-CoA (Saponaro et al., 2015). Alternatively, fructose-derived DHAP may be converted into glycerol 3-phosphate via glyceroneogenesis. Both fatty acid-CoA and glycerol 3-phosphate are substrates for *de novo* TAG synthesis (Saponaro et al., 2015). Accumulation of lipid droplets is evident in primary rat cardiomyocytes and H9c2 cardiomyoblasts following 1 mM fructose exposure for 24 h (Daniels et al., 2021). Similarly, fructose exposure increased TAG content in H9c2 cardiomyoblasts and primary mouse cardiomyocytes (Kang et al., 2016; Fan et al., 2018). Fructose-induced cardiomyocyte lipid accumulation may in part be due to changes in gene expression, as primary mouse cardiomyocytes cultured in 5 mM fructose for 24 h exhibit increased mRNA expression of enzymes involved in *de novo* production of fatty acid-CoA, FAS and stearoyl-CoA desaturase-1 (SCD1) (Fan et al., 2018). Upregulated fructose metabolism has also been linked to cardiomyocyte lipid production, as fructokinase-C overexpression significantly increased lipid content in neonatal mouse cardiomyocytes (Mirtschink et al., 2015). Given the evidence that cardiac fructose is elevated in diabetic hearts *in vivo* and fructose exposure *in vitro* promotes cardiomyocyte lipid accumulation, a case can be made that fructose may contribute to lipid accumulation in diabetic cardiomyopathy, but to date this has not been directly investigated.

### Fructose-Derived Cardiomyocyte Post-translational Modifications

Intracellular cardiac proteins can be altered through irreversible non-enzymatic glycation modification of amino acid residues known as advanced glycation end products (AGEs). The AGE formation process is initiated by the attachment of a hexose sugar (e.g. glucose, fructose) to a protein to form a Schiff base. Schiff bases are rearranged into AGE intermediates (e.g., Amadori products) and ultimately form AGEs following a series of oxidation-based Maillard reactions (Cho et al., 2007). Production of AGEs and their interaction with receptors for AGEs (RAGEs) can contribute to contractile dysfunction, increased inflammation and oxidative stress in cardiomyocytes (Bodiga et al., 2014). The molecular mechanisms underlying the link between AGE formation and diabetic cardiac pathology are not fully elucidated but there is some indication that direct obstruction of sarcomeric proteins is involved (Papadaki et al., 2018). Clinically, increased serum AGEs are associated with impaired left ventricular diastolic function in diabetic patients (Berg et al., 1999; Sveen et al., 2014). Experimental rodent models of diabetes exhibit increased AGE modification of cardiac proteins involved in excitation-contraction coupling (Bidasee et al., 2003, 2004). Interestingly, formation of fructose-derived AGEs occurs much more rapidly than glucose-derived AGEs (McPherson et al., 1988; Suárez et al., 1989), suggesting that fructose poses a greater risk for protein modification than glucose. The open-chain conformation of fructose likely results in faster glycation kinetics, production of Heyns products (a fructose homolog to Amadori products) and glycation of proteins and lipids (McPherson et al., 1988; Suárez et al., 1989; Schalkwijk et al., 2004; Takeuchi et al., 2010). Type 1 diabetic patients have 4-fold higher circulating levels of fructose-derived AGEs relative to healthy controls (Takeuchi et al., 2010). Incubation of purified cardiac troponin−C, the Ca^2+^ binding protein in the troponin complex which regulates cross-bridge cycling, with high fructose led to dramatically higher levels of AGE formation (carboxymethyllysine) and oxidation relative to high glucose conditions (Janssens et al., 2018). In non-cardiac cells, upregulation of the polyol pathway via sorbitol dehydrogenase overexpression increased fructose-derived AGEs (Takeuchi et al., 2010). Similarly, high fructose exposure increased fructose-derived AGE formation in cultured human dendritic cells (Jaiswal et al., 2019). These studies suggest that elevated circulating fructose levels and intracellular fructose production (polyol pathway) in diabetes may have an increased capacity to produce AGEs.

Elevated intracellular fructose may also have the potential to modify cardiomyocyte function via *O*-GlcNAcylation post-translational modification of proteins. In contrast to AGE formation, *O*-GlcNAcylation is a reversible modification of serine or threonine residues, mediated by an enzymatic process involving the hexosamine biosynthesis pathway. The glycolytic intermediate, fructose-6-phosphate, provides a substrate for UDP-GlcNAc, the precursor for *O*-GlcNAcylation. *O*-GlcNAcylation has been identified as an important mediator of cardiac pathologies (McLarty et al., 2013; Marsh et al., 2014) that may play a role in the development of diabetic cardiomyopathy (Hu et al., 2005; Erickson et al., 2013; Prakoso et al., 2021). Elevated levels of *O*-GlcNAcylation have been observed in left ventricular tissues of fructose-fed rodents (Hecker et al., 2012), but the direct involvement of fructose in the hexosamine biosynthesis pathway and *O*-GlcNAcylation in the heart has not been investigated. Evidence from non-cardiac cell lines indicates that fructose can promote *O*-GlcNAcylation, as increased UDP-*O*-GlcNAc levels are observed in HepG2 (liver) cells exposed to fructose for 24 h (Hirahatake et al., 2011). Together these studies suggest that fructose-derived *O*-GlcNAcylation is a potentially important signaling route.

### Fructose-Induced Cardiomyocyte Inflammation, Cell Death, and Pathological Growth

Emerging evidence suggests that activation of inflammatory pathways is an important mediator of cardiac pathology in diabetes (Frati et al., 2017). *In vitro* studies have shown that cardiomyocyte fructose exposure upregulates the expression of pro-inflammatory cytokines, a hallmark of cellular stress (Kang et al., 2016; Zhang et al., 2016a; Lian et al., 2017; Xie, 2017; Zhao et al., 2017; Fan et al., 2018). Increased cytokine expression in this context is coupled to upregulated pro-inflammatory nuclear factor-κB (NF-κB) and mitogen-activated protein kinases (MAPKs) pathways (Table 1), partly via interleukin-1 receptor-associated kinase 4/1 (IRAK4/1) and nucleotide-binding domain (NOD)-like receptor protein 4 (NLRP4) (Zhang et al., 2016a; Kang et al., 2016; Lian et al., 2017; Xie, 2017; Zhao et al., 2017). Fructose-induced cardiac inflammation may also be associated with the production of reactive oxygen species (ROS), and fructose exposure has been shown to increase ROS and suppress antioxidant defense systems in cultured cardiomyocytes (Zhao et al., 2017; Fan et al., 2018; Park et al., 2018). Upregulated cardiac inflammation and ROS production may underlie fructose-induced increase in apoptotic indices in primary mouse cardiomyocytes (Fan et al., 2018) and H9c2 cardiomyoblasts (Zhao et al., 2017; Park et al., 2018), coincident with decreased cell viability (Zhao et al., 2017). Interestingly, a glucagon-like peptide-1 (GLP-1) agonist has been shown to ameliorate fructose-induced cell death in cultured neuronal cells (Khalilnezhad and Taskiran, 2019), but to date this has not been investigated in cardiomyocytes. GLP-1 agonists are an emerging class of T2D therapies and the modes of action involve signaling, metabolic and functional pathways (Rowlands et al., 2018). Investigation into a role for GLP-1 signaling in regulating fructose cardiomyocyte toxicity would be informative. Inflammatory cytokines have also been linked to the development of cardiac hypertrophy and fructose exposure *in vitro* increases the production of tumor necrosis factor (TNF)-α, interleukin (IL)-1β, IL-6 and transforming growth factor (TGF)-β1 in cardiomyocytes (Zhang et al., 2016a; Kang et al., 2016; Lian et al., 2017; Xie, 2017; Zhao et al., 2017; Fan et al., 2018). These cytokines are suggested to influence cardiac growth through upregulation of growth-related signaling pathways such as MAPK-JNK and CaMKII-STAT3 (Thaik et al., 1995; Condorelli et al., 2002; Matsumoto-Ida et al., 2006; Zhang et al., 2016a; Zhao et al., 2016). *In vitro,* high fructose exposure (25–50 mM) increased cell size in H9c2 cardiomyoblasts and neonatal rat ventricular myocytes (Zhang et al., 2016b; Park et al., 2018), linked with increased expression of hypertrophic markers (Park et al., 2018). Thus it is evident that fructose exposure *in vitro* promotes cardiomyocyte inflammation, cell death and pathological growth.

**TABLE 1:** Fructose-induced cardiomyocyte pathology.

Cell type	Fructose concentration	Treatment duration (hr)	Cardiomyocyte pathology	Reference
H9c2	1 mM	24	↑ Inflammation	Kang et al. (2016)
			↑ Fat storage	
AMC	2 mM	24	↑ Inflammation	Zhang et al. (2016a)
			↑ ROS production	
			↑ NF-κB signaling	
			↑ p38 MAPK signaling	
NRVM	25 mM	48	↑ Hypertrophy	Zhang et al. (2016b)
			↑ Mitochondrial-derived ROS	
H9c2	5 μM	24	↑ Inflammation	Xie, (2017)
			↑ Collagen	
			↑ NF-κB signaling	
H9c2 and HL-1	5 mM	24	↑ Inflammation	Lian et al. (2017)
			↑ NF-κB signaling	
H9c2	5 mM	24	↓ Cell viability	Zhao et al. (2017)
			↑ Inflammation	
			↓ Antioxidant levels	
			↓ AMPK activity	
			↑ NF-κB signaling	
H9c2	50 mM	24	↑ Hypertrophy	Park et al. (2018)
			↓ Mitochondrial respiration	
			↑ Mitochondrial-derived ROS	
			↑ Apoptosis	
			↓ AMPK activity	
AMC	5 mM	24	↑ Fat storage and synthesis	Fan et al. (2018)
			↑ Lipid oxidation	
			↑ ROS production	
			↑ Inflammation	
			↑ NF-κB signaling	
NRVM and H9c2	1 mM	24	↑ Fat storage	Daniels et al. (2021)

Rat cardiomyoblast cell line (H9c2), mouse atrial cell line (HL-1), adult mouse cardiomyocyte (AMC), neonatal rat ventricular myocyte (NRVM), reactive oxygen species (ROS), nuclear factor kappa-light-chain-enhancer of activated B cells (NF-κB), mitogen-activated protein kinase (MAPK), AMP-activated protein kinase (AMPK).

## Conclusion and Future Perspectives

Emerging evidence suggests that elevated fructose exposure and upregulated fructose metabolism may have significant impact on cardiomyocytes. Recent findings that diabetic patients exhibit elevated cardiac fructose and sorbitol content provide an important platform for ongoing work to understand the consequences of elevated fructose in the heart. *In vitro* studies have demonstrated that cardiomyocyte fructose exposure is linked with metabolic disturbance, lipid accumulation, inflammation, apoptosis and pathological growth. Further, intracellular fructose has the potential for post-translational modification of proteins, which may constitute an important mechanistic pathway for fructose-mediated disturbances in cardiomyocyte signaling and function. Significant knowledge gaps remain, and future investigations translating the key *in vitro* findings to an *in vivo* context are now warranted. Intervention studies targeting fructose-related pathways in the heart (e.g., fructose transport, fructose metabolism, the polyol pathway, fructose-derived AGEs) will provide important information on the potential role of fructose in mediating cardiac pathology. Fructose metabolism may prove to be an effective therapeutic target to mitigate the metabolic disturbances evident in diabetic heart disease, and understanding the role of fructose in the heart, in health and in disease states, is an important priority.
